# Melatonin May Increase Anticancer Potential of Pleiotropic Drugs

**DOI:** 10.3390/ijms19123910

**Published:** 2018-12-06

**Authors:** Bianka Bojková, Peter Kubatka, Tawar Qaradakhi, Anthony Zulli, Karol Kajo

**Affiliations:** 1 Department of Animal Physiology, Institute of Biology and Ecology, Faculty of Science, Pavol Jozef Šafárik University in Košice, Šrobárová 2, 041 54 Košice, Slovak Republic; 2 Department of Medical Biology, Jessenius Faculty of Medicine, Comenius University in Bratislava, Malá Hora 4, 036 01 Martin, Slovak Republic; Peter.Kubatka@jfmed.uniba.sk; 3 Department of Experimental Carcinogenesis, Division of Oncology, Biomedical Center Martin, Jessenius Faculty of Medicine, Comenius University in Bratislava, Malá Hora 4C, 036 01 Martin, Slovak Republic; 4 Institute for Health and Sport (IHES), Victoria University, Melbourne, VIC 3011, Australia; tawar.qaradakhi@live.vu.edu.au (T.Q.); anthony.zulli@vu.edu.au (A.Z.); 5 St. Elisabeth Oncology Institute, Heydukova 10, 811 08 Bratislava, Slovak Republic; karol.kajo@ousa.sk; 6 Biomedical Research Center, Slovak Academy of Sciences, Dúbravská cesta 9, 845 05 Bratislava, Slovak Republic

**Keywords:** melatonin, cancer, NSAIDs, statins, antidiabetics

## Abstract

Melatonin (N-acetyl-5-methoxytryptamine) is not only a pineal hormone, but also an ubiquitary molecule present in plants and part of our diet. Numerous preclinical and some clinical reports pointed to its multiple beneficial effects including oncostatic properties, and as such, it has become one of the most aspiring goals in cancer prevention/therapy. A link between cancer and inflammation and/or metabolic disorders has been well established and the therapy of these conditions with so-called pleiotropic drugs, which include non-steroidal anti-inflammatory drugs, statins and peroral antidiabetics, modulates a cancer risk too. Adjuvant therapy with melatonin may improve the oncostatic potential of these drugs. Results from preclinical studies are limited though support this hypothesis, which, however, remains to be verified by further research.

## 1. Introduction

**Figure 1:**
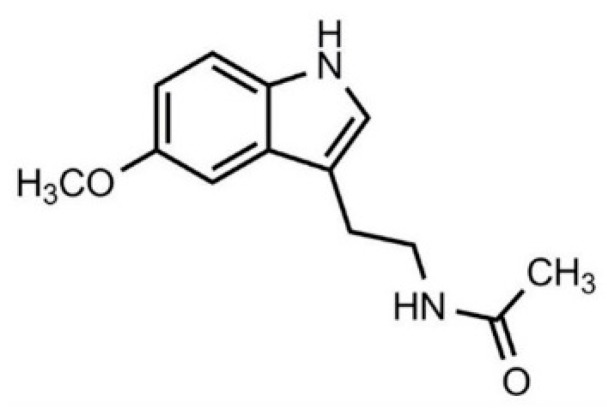
Structure of melatonin (MEL).

Prevention and/or delay of progression of cancer are great challenges for oncologists. Cancer incidence is higher in older subjects, which can be a result of hormonal imbalance and metabolic dysregulation, and is the leading cause of worldwide morbidity and mortality [1]. Numerous drugs have been shown to inhibit malignant transformation in preclinical research, however, the transfer of these results to clinical practice remains complicated. A possible approach is to focus on drugs that are primarily used for therapy of other diseases but also exert oncostatic properties, which may be potentiated in combination with other oncostatic substances, preferably those with minimum toxicity. Melatonin (MEL (Figure 1)), a derivate of amino acid tryptophan, is an ubiquitous molecule with numerous beneficial effects including being oncostatic [2,3,4,5]. Clinical results showed that chemotherapy in combination with MEL supplementation increased survival in patients with various solid tumors [6]; however, long-term epidemiological studies are needed as MEL efficacy in humans is yet to be determined. Nonetheless, preclinical evidence regarding the effects of MEL in cancer [7], but also in a number of other pathologies, including metabolic, cardiovascular and neurodegenerative disorders [8] is promising. In addition, MEL has been reported to increase the efficacy and reduce the toxicity of numerous drugs [9,10]. Overcoming drug resistance in chemo/radiotherapy of various cancers is of particular interest [11]. Considering its excellent safety profile [12], it seems plausible that among many other options, adding MEL to the therapy of common diseases of (but not limited to) older age, arthritis, dyslipidemia and type 2 diabetes might be useful in cancer management. In this review, we summarized the preclinical and clinical results regarding the oncostatic efficacy of MEL and three groups of pleiotropic drugs, non-steroidal anti-inflammatory drugs (NSAIDs), statins and peroral antidiabetics, together with available data on their combination.

### Source of Data

Data from the biomedical literature were collected and analyzed. Relevant studies published almost exclusively in the English-language literature were retrieved by the use of “melatonin” or “cancer” or “cell lines” or “mechanism of action” or “non-steroidal anti-inflammatory drugs” or “coxibs” or “statins” or “antidiabetics” or “metformin” or “glitazones” or “thiazolidinediones” or “retinoids” or “treatment” or “chemoprevention” or “animal model” or “clinical trials” as either a keyword or MeSH (medical subject heading) term in searches of the PubMed bibliographic database. We focused primarily on the most recent scientific papers from the years 2013–2018.

## 2. MEL—A Versatile Molecule

From the identification in the bovine pineal gland in 1958 [13], and later in extrapineal sites [14], MEL synthesis was long believed to be restricted to vertebrates. MEL was then identified in the eyes of the non-vertebrate, locust, in 1984 [15], and since then, MEL has been detected in other taxa too, including unicellulars, bacteria and fungi [16]. The production of MEL in mammals is not restricted to the pineal gland; synthesis takes place in other organs including the retina, gastrointestinal tract, and reproductive tract; however, the functions of extrapineal MEL are not yet fully understood [17].

MEL synthesis in plants is only evidenced from 1995 [18] and it was already well-known that, like in animals, phytoMEL (the structure of plant and animal MEL are same) displays a large set of functions including regulation of growth and development and response to abiotic/biotic stressors [19,20,21]. A circadian rhythm of MEL seems to also exist in plants [22].

The effect of MEL in mammals reaches far beyond its role as a chronobiotic. A vast number of reports point to cytoprotective efficacy of MEL in multiple taxa, which is contributed, but not restricted to antioxidant effects [23]. The protection of normal cells from oxidative stress and pro-oxidant activity in cancer cells [24], together with other properties including cell cycle regulation, pro-apoptotic, anti-metastatic, antiangiogenic and immunomodulatory activity, stand behind the anti-cancer effects of this indolamine [25].

## 3. Sources of MEL

In most species, the pineal gland, a secretory organ, is the major source of MEL production in response to darkness [26,27]. It has been established that the orbital associated organs such as the eye (retina and iris), Harderian gland and lacrimal gland can also produce MEL [28,29], as well as serotonin-rich entero-endocrine cells of the gastrointestinal mucosa [30].

Although MEL is produced within the body, it can also be obtained from animal foods, edible or medicinal plants and MEL supplements. Once exogenous MEL is consumed, it gets absorbed by the gastrointestinal tract (GIT) and then enters the circulation.

Quantified by High Performance Liquid Chromatography (HPLC), MEL content in animal foods, meat (lamb, beef, pork), fish (salmon) and chicken, was found to range from 1 ng/g to 3.7 ng/g [31]. These ranges are higher than the MEL concentration found in the serum of healthy individuals (reported to range from 0.05 to 0.2 ng/g) [32], suggesting that a dietary intake of these animal foods could possibly increase MEL in humans when required.

In 1995, it was discovered that MEL is also found in plants, including fruits, vegetables, seeds and nuts [33]. PhytoMEL is structurally identical to animal and synthetic (supplementary) MEL, and therefore, intake of phytoMEL should exert the same effect [33].

MEL in the form of powder and tablets are designed for conditions in which MEL supplementation is recommended, however, factors including insufficient absorption (possibly due to poor solubility) or conversion of MEL before entering the circulation at the hepatic system can influence the bioavailability and its optimal effect during treatments [34,35]. Various techniques have been used to assess the drawbacks of raw/oral MEL; for example, recently, a group in Italy studied the bioavailability of MEL in gel capsules and found that compared to powdered MEL, the capsule improved serum bioavailability of MEL in humans [36]. Other techniques include melt crystallization technique to enhance solubility of MEL [37] and nanotechnology, where MEL is encapsulated into a nano-matrix called a nanosphere for sustained release of MEL [38,39].

### 3.1. MEL in Animal Foods

There is limited research regarding MEL in animal foods [40]; however, Tan et al. [31] reported that MEL is found in animal products and in cow’s milk at 0.014 ng/g [31,41,42], human breast milk, containing up to 0.042 ng/g [31,43] and in the colostrum [31]. Interestingly, the concentration of MEL was found to be higher in fish sources (specifically salmon at 3.7 ng/g) and eggs (6.1 ng/g), compared to other animal products including beef (2.2 ng/g), lamb (1.6 ng/g), pork (2.5 ng/g) and milk [31].

In summary, it is likely that MEL levels can increase after the consumption of animal foods rich in MEL. Future studies are required to confirm the relationship between animal food intake and serum MEL concentrations/bioavailability in humans.

### 3.2. MEL in Edible and Medicinal Plants

There are a number of studies on MEL and its concentration in a variety of edible and medicinal plants. A recent review by Bonomini [44] provided a succinct description of most of the plants and their associated MEL concentrations. The average concentration of fruits such as banana, cucumber, tomatoes and beetroot is 0.47 ng/g, 0.09 ng/g, 0.25 ng/g and 0.009 ng/g, respectively [44]. Interestingly, MEL concentration is much more abundant in rice compared to fruits ranging from 73 to 207 ng/g [44,45]. MEL in nuts has also been investigated, a group in America determined through HPLC that the average MEL content in walnuts (*Juglans regia* L.) is 3.5 ng/g, and this concentration is enough to influence serum MEL levels in rats [46], suggesting that the intake of nuts alone may be beneficial in increasing MEL levels.

Moreover, MEL is found in medicinal plants that have been used for centuries. The Mediterranean shrub (buckthorn) and subshrub (sage), as well as laurel, were found to have MEL levels from 0.3 to 8 ng/g within the leaves and associated fleshy fruits [47,48]. However, Chinese medicinal herbs, such as *Viola philipica Cav*, *Uncaria rhynchophylla Mig* and *Phellodendron amurens*, exhibit much higher MEL concentrations, at 2368 ng/g, 2460 ng/g 1235 ng/g, respectively [49], compared to the Mediterranean herbs and edible plants. Furthermore, MEL content in plants varies greatly [40], not only among various species, but also within one species depending on environmental factors. Abiotic stressors as chemicals [50], high temperature [51] or UV radiation [52] were reported to increase its content in plants.

### 3.3. MEL Supplements and Bioavailability

MEL is a general supplement that can be purchased both over the counter and with a prescription from the pharmacy. It comes in various formulations including tablets, gel capsules and nanospheres [53]. The bioavailability of oral MEL (2 mg and 4 mg) is only 15% in the circulation, suggesting that 85% of oral MEL is metabolized by large first pass metabolism or due to poor oral absorption [34]. To combat the large first pass metabolism, gel capsules have been designed to improve the bioavailability. Previously, a study reported that 1 mg of MEL encapsulated within a soft gel capsule takes the same time as a 1 mg oral MEL (in powder form) to reach maximum peak in the plasma of healthy individuals. However, measured as Area Under the Curve (AUC), MEL bioavailability was markedly increased from the soft gel capsule compared to the 1 mg oral MEL [36]. Further studies are required to assess the gel capsule MEL compared to commercial MEL to confirm these promising results.

Over 30 oral MEL supplements were analyzed by liquid chromatography for detection of MEL and serotonin content and found that 26% of the oral supplements contained serotonin [53], suggesting that MEL supplements are not free of contaminants and may affect other physiological factors. To address this, a group in China proposed a method using nanotechnology whereby silicon dioxide as a nanosphere coated with hydroxypropyl methylcellulose phthalate was used as MEL carrier in rats, ensuring that it was only MEL being absorbed upon administration [39]. The nanosphere increased maximum peak concentration of MEL in the plasma and increased the AUC compared to commercial oral MEL in rats [39]. Future studies are required to determine the effects of nanosphere MEL in animal studies followed by the pharmacokinetic parameters in humans to establish whether it can be beneficial in releasing MEL into the bloodstream with improved bioavailability.

## 4. MEL and Cancer and Vice Versa

Disturbances in MEL secretion may contribute to cancer risk and it has been reported that MEL secretion in patients with breast, endometrial and colon cancer is impaired [54]. Apart from physiological decline with age, nocturnal serum levels of MEL are mainly reduced by artificial light exposure at night, which leads to disruption to the circadian system, with alterations of sleep-activity patterns, suppression of MEL production, and deregulation of circadian genes involved in cancer-related pathways [55]. The relationship between continuous exposure to light at night, known as functional pinealectomy, and higher cancer rates is long well-known from animal studies and MEL was proven to reverse this effect of light [56,57]; however, human data have not been cohesive. In addition, most experimental and human data concerns mammary cancer. Several studies reported increased risk of breast [58,59], colorectal [60] and prostate cancer [61,62] in night shift workers, however, according to others [63,64], no relation can be confirmed. The large differences in definitions of both exposure and outcome may contribute to the observed heterogeneity of the results [65]; therefore, additional standardized studies are needed to improve the epidemiologic evidence. Nevertheless, exogenous MEL may compensate for disturbances in endogenous production and subsequent adverse consequences. In the next chapters, the mechanisms of oncostatic activity of MEL and results of preclinical and clinical studies are summarized.

### 4.1. Mechanism of Antitumor Activity of MEL

It has been documented that the disruption of the circadian nocturnal melatonin signal promotes the consequent signaling, including the metabolism, initiation and progression, of carcinogenesis [66]. Circadian genes demonstrate clock functions that modulate the expression of numerous genes with circadian rhythmicity, which are linked with the daily oscillations of proteins. In this regard, the disruption in the circadian organization of these genes and related protein expressions leads to deregulated cell proliferation and subsequent tumorigenesis. Additionally, circadian genes possess non-clock activities, which are crucial in the processes related to cancer, such as cell cycle progression, DNA damage response and genomic stability, and drive cancer cells to endocrine and chemotherapeutic resistance [67]. Growing evidence suggests that prolonged shift work and other activities during night may negatively impact circadian rhythms and lead to multi-system disease, including cancer [68]. Therefore, circadian rhythm disruption may play an important role in cancer biology [67,69].

**Figure 2:**
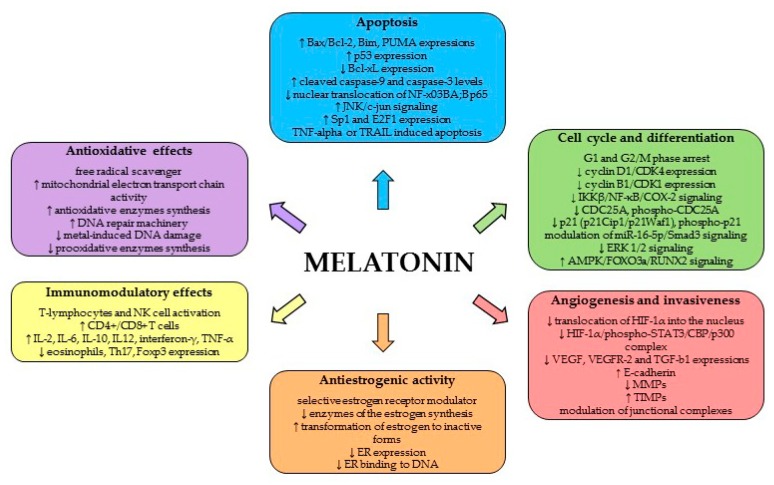
Oncostatic mechanisms of action of MEL.

In addition to the timekeeping function of MEL, the pleiotropic functions of this molecule involve numerous physiological processes, including anticancer activity. MEL is an effective antioxidant, and is characterized by an apparent pro-apoptotic signaling function and the modulation of cell cycle and differentiation. Moreover, MEL demonstrates anti-metastatic and antiestrogenic properties and immunomodulatory effects (review [70] (Figure 2)).

#### 4.1.1. Antioxidant Effects

It has been well-described that MEL and its metabolites manifest significant antioxidative effect, providing protection against DNA damage from mutagenic molecules and behaving as an effective free radical scavenger [71,72]. Recent reviews by Galano et al. [73] and Reiter et al. [5] concluded that MEL antioxidant action also includes indirect mechanisms; they comprise enhancing the activity of mitochondrial electron transport chain, inhibition of metal-induced DNA damage, protection against non-radical triggers of oxidative DNA damage, continuous protection after being metabolized, activation of antioxidative enzymes (such as glutathione), inhibition of pro-oxidative enzymes and boosting of the DNA repair machinery.

#### 4.1.2. Apoptosis, Cell Cycle and Differentiation

Among the main direct anticancer mechanisms of MEL belong pro-apoptotic, antiproliferative, differentiating and anti-angiogenic mode of action. MEL has been described as a molecule activating caspases through the intrinsic, mitochondrial-dependent mechanisms and increasing the Bax/Bcl-2 ratio and p53 expression, which lead to programmed cell death [74,75]. The role of MEL in the apoptosis of cancer cells seems to be attractive for oncology research, because it promotes apoptotic processes in most cancer cells, in contrast to the obvious inhibition of apoptosis in normal cells [76]. MEL is capable to induce apoptosis itself [74,77,78,79], even though not all studies have been able to confirm this effect [80]; however, combined treatment increases the pro-apoptotic effects of MEL in cancer cells [81,82,83].

The tumor suppressive effects of MEL have also been attributed to the reduction of cancer promotion or progression, which are associated with antiproliferative activities. It has been well-described that the antiproliferative properties of MEL take place through cell cycle arrest [84,85,86]. Moreover, MEL’s ability to decrease cancer cell proliferation has been ascribed to enhancing of phosphoactivation and transactivation of a number of transcription factors and nuclear binding sites that are involved in the modulation of carcinogenesis [5,87]. MEL’s differentiating properties against solid and liquid tumors have also been documented (review of Di Bella et al. [4]). MEL decreases prostate cancer cell growth leading to neuroendocrine differentiation via a receptor and protein kinase A (PKA) independent mechanism [88]. In breast cancer, MEL stimulates the differentiation of fibroblasts and downregulates the aromatase activity and expression in both fibroblasts and adipocytes, resulting in the suppression of estrogen-producing cells proximal to malignant cells [89]. Gastric adenocarcinoma cell line SGC-7901 treated with MEL showed more differentiated morphologic phenotype when compared to untreated cells [90], suggesting that MEL acts as a differentiation inducer.

#### 4.1.3. Angiogenesis and Invasiveness

MEL may also exert its anticancer effects also through angiogenesis inhibition. Hypoxia induced factor-1α (HIF-1α) and the genes controlled by HIF-1a, such as the vascular endothelial growth factor (VEGF), are the important molecular targets of MEL in the angiogenesis inhibition. MEL blocks the translocation of HIF-1α into the nucleus thereby suppressing VEGF expression and reduces the formation of HIF-1α, phospho-STAT3 and CBP/p300 complex, which is the key regulator of the angiogenesis-related genes expression [91]. Moreover, the anti-angiogenic effect of MEL was described in an animal model of ethanol consumption, where MEL attenuated HIF-1a, VEGF, and transforming growth factor-b1 expression in ovarian cancer [92]. Through the downregulation of VEGF, MEL confirmed anti-angiogenic effects in SH-SY5Y neuroblastoma cells [93] and MDA-MB-231 xenograft model of breast carcinoma [94].

#### 4.1.4. Antiestrogenic Activity

MEL, through its antiestrogenic and antigonadotropic actions, behaves as an anti-tumor substance, predominantly in hormone-dependent breast tumors. There are numerous experimental data proving the obvious interference of MEL with the modulation of estrogen receptor (ER) activity [87] with the production of estrogens via the inhibition of the enzymes involved in the synthesis of estrogen [95,96], and with the metabolism of estrogens through the transformation of estradiol into inactive estrogen sulphate/sulfate form [97]. MEL behaves as selective ER modulator (SERM) that downregulates the expression of ER and also weakens the ER binding to DNA [98].

#### 4.1.5. Immunomodulatory Effects

Pleiotropic effects of MEL in organisms include immunomodulatory effects. While some investigators described MEL as an immuno-stimulant, many other studies have also argued anti-inflammatory activities (review of Carrillo-Vico et al. [99]). MEL is involved in the regulation of both cellular and humoral immunity. The fundamental physiological role of MEL on the immunity has been well-described. The immunomodulatory properties of MEL are mediated through the activated T-lymphocytes via opiatergic mechanism [100] or enhanced immunity mediated by CD8+ T cells [101]. MEL has been shown to increase T-helper cell activity by releasing several specific cytokines [102]. MEL’s oncostatic actions include the direct augmentation of natural killer cell, monocytes and leukocytes activity, which increases immunosurveillance, as well as the stimulation of cytokine production, such as interleukin-2, interleukin-6, interleukin-10, interleukin-12 and interferon-gamma by the mononucleate cells [4,54]. Most recent study pointed to MEL’s immunomodulatory activities through the suppression on eosinophils and Th17 cells and Foxp3 expression, on the other hand, enhancing of CD4+ cells and TNF-α [103].

### 4.2. Preclinical Studies

MEL could be an excellent candidate for the prevention and treatment of several cancers, such as breast, ovarian, prostate, gastric, colorectal, pancreatic, liver, renal or lung. Numerous preclinical studies have aimed to evaluate the anticancer activity and mechanisms of action of MEL. Moreover, MEL analogs developed in recent years and tested for their role in the prevention or treatment of neoplastic disease [104,105,106,107] may initiate a whole new era in cancer research.

#### 4.2.1. MEL and Cancer: In Vitro

Using MEL as an anticancer drug, many cancer types have been analyzed and multiple modes of action have been proposed. Among the most studied cancer type belongs breast carcinoma. In estrogen receptor-positive (ERα+) human breast cancer cells, MEL downregulated both ERα mRNA expression and estrogen-induced transcriptional activity of the ERα through MEL receptors 1 (MT 1)-induced activation of G(αi2) signaling with the consequent reduction of cAMP levels [108]. Additionally, MEL regulates the transcription of additional members of the nuclear receptor super-family which play an important role in cancer signaling [109]. The anti-invasive effects of MEL include the blockade of p38 phosphorylation [82] and the expression of matrix metalloproteinases [110,111]. In addition, MEL’s anticancer mode of action includes the modulation of cell viability and angiogenesis and inflammation in triple-negative breast cancer cell line (MDA-MB-231) [112].

In ovarian cancer, MEL possess both membrane and intracellular mode of actions that lead to the inhibition of cell proliferation, angiogenesis, migration and anti-cancer stem cells (CSC) activity. This regulation may directly involve intracellular targets or it may occur indirectly via MT_1_ receptors [113]. MEL showed antiproliferative activity in two ovarian cancer cell lines (OVCAR3 and SKOV3) via inhibition of ERα expression [114]. In another study, MEL induced a marked increase in E-cadherin along with decrease in VEGF expression levels in SKOV3 cell line. This result determines the anti-invasive activities of this indoleamine [115]. Akbarzadeh et al. [116] evaluated invasiveness and migration of cancer stem cells (CSCs) isolated from SKOV3 cells. MEL inhibited epithelial mesenchymal transition related gene expressions including ZEB1, ZEB2, snail and vimentin with increase in E-cadherin. MEL treatment showed an apparent decrease in the expression and activity of matrix MMP-9 in CSCs. Finally, MEL inhibited migration of CSCs in a partially receptor dependent and PI3k and MAPK independent manner.

In prostate cancer, LNCaP and 22Rv1 prostate cancer cells transiently overexpress androgen receptor splice variant-7 (AR-V7), and consequently activate the nuclear factor-kappa B (NF-κB) and upregulate interleukin (*IL*)*-6* gene expression. MEL inhibited NF-κB activation through MT₁ receptor-mediated antiproliferative pathway, and can disrupt bi-directional positive interactions between AR-V7 and NF-κB in prostate cancer cell lines. Through this mechanism, MEL delays the development of castration resistance in advanced prostate cancer [117]. In another study, MEL blocked nuclear translocation of androgen receptor in LNCaP cells, and thus, confirmed anti-androgenic mode of actions. Moreover, the authors found that IGFBP3 and MAPK/ERK signaling mediate MEL-induced anticancer effects in prostate cancer cells [118]. Another in vitro study showed that MEL decreased the expression of hypoxia-inducible factor (HIF)-1 alpha, HIF-2 alpha, and vascular endothelial growth factor (VEGF) at mRNA level in hypoxic PC-3 prostate cancer cell line [119]. Further evaluation showed that upregulation of miRNA3195 and miRNA374b regulated the anti-angiogenic property induced by MEL in hypoxic PC-3 cells [119]. Finally, MEL promoted phenotypic changes making prostate cancer cells more sensitive to cytokine mediated apoptosis (via TNF-alpha or TRAIL) [120] or is able to induce positive epigenetic changes in these cells [121].

MEL causes cell cycle arrest and suppression of CDC25A, phospho-CDC25A (at Ser75), p21 (p21Cip1/p21Waf1) and phospho-p21 (at Thr145) expressions in SGC-7901 gastric cancer cell line [74]. In the same study, MEL showed the involvement of the mitochondria in MEL-induced apoptosis (upregulation of Bax, downregulation of Bcl-xL, an increase in cleaved caspase-9 and caspase-3 levels). All these anticancer activities were regulated through the blockade of the AKT/MDM2 signaling pathway. In addition, MEL was described as an inductor of apoptosis in AGS gastric cancer cells by activating the caspase-dependent apoptotic pathway and by suppressing the nuclear translocation of NF-x03BA;B p65, two processes that are controlled by p38 and JNK [122]. In another in vitro study, MEL suppressed the proliferation of gastric cancer cells via modulation of the miR-16-5p/Smad3 signaling pathway [123]. Wang et al. [124] pointed to the involvement of nuclear receptor RZR/RORγ in MEL-induced suppression in HIF-1α accumulation and VEGF generation in SGC-7901 human gastric cancer cells under hypoxic conditions. Finally, MEL acts as a differentiation inducer [90] and inhibits cell migration [125] in gastric cancer cells in vitro.

MEL demonstrated anticancer potential against colorectal cancer in vitro by the downregulation of endothelin-1 expression via the FoxO-1/NF-κβ signaling pathway [126]. Pro-apoptotic effects of MEL were analyzed in LoVo colorectal cancer cells. MEL-induced apoptosis was dependent on the nuclear import of HDAC4 and subsequent H3 deacetylation of the Bcl-2 promoter via the inactivation of CaMKIIα in this in vitro study [78]. Recent studies demonstrated that MEL promotes apoptosis via the inhibition of cellular prion protein expression [127,128].

In pancreatic cancer, MEL suppressed the activity of NF-κB p65 and stimulated the mitogen-activated protein kinase pathways (c-jun N-terminal kinase/extracellular-regulated kinase 1/2), which increased Bax/Bcl-2 ratio and caspase-3 cleavage in MIA PaCa-2 pancreatic carcinoma cell line [129]. Using MiaPaCa-2, AsPc-1 and Panc-28 cancer cells, MEL inhibited proliferation and invasion in a receptor-independent manner, but also overcame gemcitabine resistance in PDAC cells [130]. Most recently, MEL enhanced the efficacy of sorafenib against pancreatic cancer by downregulation of PDGFR-β/STAT3 cell signaling and MEL receptor (MT)-mediated STAT3 in PDAC cells [131].

The combinational use of standard chemotherapy with some natural compounds such as MEL may provide a potential option to improve clinical efficacy and reduce side effects within cancer treatment. In this regard, MEL sensitized the cisplatin-mediated growth suppression of liver cancer through the targeting of NF-κB/cyclooxygenase (COX)-2 and AP-2β/hTERT signaling pathway in hepatocellular carcinoma cells [132]. Similarly, MEL increased sorafenib-induced apoptosis via synergistic activation of the JNK/c-jun pathway [133] or through reactive oxygen species production in mitochondria and mitophagy in the HCC cell line [134].

Renal cell carcinoma demonstrates the highest metastasis potential among urological malignancies. MEL at the pharmacologic concentration (0.5–2 mM) significantly suppressed the migration and invasion of Caki-1 and Achn renal carcinoma cells via regulation of Akt-MAPKs cell signaling and NF-κB DNA-binding activity. These results were accompanied with the downregulation of MMP-9 by reducing p65- and p52-DNA-binding activities [135]. Another in vitro study showed that MEL increases apoptosis in Caki cells through Bim mRNA expression increase and the induction of Sp1 and E2F1 expression and transcriptional activity [136]. Furthermore, combined treatment of human renal cancer cells with MEL plus thapsigargin induced increased apoptosis when compared with thapsigargin alone. This activity was linked with ROS-mediated upregulation of CCAAT-enhancer-binding protein homologous protein [137].

Several in vitro studies pointed to the anticancer potential of MEL against lung cancer. Lu et al. [138] concluded that MEL increased the tumor suppressive effects of berberine via the inhibition of cell proliferation and migration and increased apoptosis. These changes were associated with the activating caspase/Cyto C and inhibiting AP-2β/hTERT, NF-κB/COX-2 and Akt/ERK cell signaling [138]. In vitro-administered MEL significantly decreased the viability of human A549 and PC9 lung adenocarcinoma cells. The same study revealed that MEL reduced cell adhesion, migration and the intracellular glutathione level and increased apoptosis via the increasing of caspase 3, PUMA and Bax activity and reactive oxygen species in the cells and through decreasing of PCNA and Bcl-2 activity [139]. Moreover, MEL targeted HDAC signaling in lung adenocarcinoma cells [139]. Recently, MEL has been described as a molecule that interrupts PARP-1 interaction with the telomeric long noncoding RNA (lncRNA) or chromatin, and thus, controls the senescence-associated secretory phenotype in human fetal lung fibroblast cells [140].

The anticancer effect of MEL in vitro has also been observed in cervical [141,142], melanoma [143,144], osteosarcoma [145,146], glioblastoma [147,148] or leukemia cancer cell lines [149,150,151].

#### 4.2.2. MEL and Cancer: In Vivo

In 1959, Wurtman et al. reported that bovine pineal extracts reversed the hypertrophy of the pituitary, the adrenals and the ovaries induced by pinealectomy in rats [152]. The first published evidence of the involvement of pineal gland in carcinogenesis was in 1963, when it was shown that pinealectomy accelerates the growth and spread of Walker 256 carcinoma in rats [153]. The research interest in pineal gland and MEL grew, and in 1973, two in vivo reports pointed to the antitumor activity of MEL, the first by Anisimov et al. who evaluated its effects together with extracts from the epiphysis and hypothalamus in mice with transplantable mammary tumors [154] and the second by El-Domeiri and Das Gupta, who reported the reversal by MEL of the effect of pinealectomy on melanoma transplants growth in hamsters [155]. Since then, various animal models, predominantly rodents, have been used to determine the oncostatic effects of MEL, particularly in mammary cancer. It was not the aim of this paper to bring these data in detail; these have been covered in several excellent reviews [5,7,156,157]. Most of them confirmed the inhibition of tumor growth through different mechanisms, including apoptosis induction, cell cycle and epigenetics regulation, antioxidant activity, modulation of immunity and tumor microenvironment and regulation of angiogenesis [5]. MEL enhanced chemotherapy-induced toxicity in cancerous cells through increasing apoptosis, oxidative stress and mitochondrial malfunction also alleviated side-effects of chemotherapeutics, including reproductive injury [9,98,158,159,160]. An overcome of radioresistance after MEL administration was reported too [161]. The disappointing fact to remember, however, is that none of the in vivo model (chemically-induced tumorigenesis, xenografts, genetically-engineered or animals with spontaneous cancer) can fully reproduce human cancer. In addition, experimental protocols differ greatly regarding the time, manner, dose and route of administration of MEL. Relevant proof of MEL oncostatic relevance can be obtained only from standardized human studies.

#### 4.2.3. MEL and Cancer: Clinical Results

The research concerning MEL used as an adjuvant to chemotherapy in cancer patients has been initiated by Lissoni group in the 1980s. Most of the trials evaluated MEL effects (administered per os for several weeks/months) as an adjuvant to standard chemotherapy; the doses were supraphysiological, ranging in tenths of mg per day. To compare, a typical dose of commercially available peroral MEL used to treat jet-lag or sleep disorders ranges from 3–5 mg. In patients with metastatic solid tumors (breast cancer, non-small cell lung carcinoma, gastrointestinal and head and neck carcinoma), MEL reduced the toxicity and enhanced the effect of standard chemotherapy resulting in tumor regression and increased survival time [162,163,164,165]. MEL also prolonged survival in patients with advanced primary hepatocellular carcinoma [166], melanoma [167] and glioblastoma patients treated with radiotherapy [168]. The effect of immunomodulator IL-2 on solid tumors (breast cancer, non-small cell lung carcinoma, gastrointestinal cancer) was potentiated by co-administration with MEL as evidenced by the increase in tumor objective regression rate and survival [169,170]. MEL enhanced the effect of chemotherapy in patients with colorectal metastatic cancer, the percent of disease-control achieved in patients concomitantly treated with MEL was significantly higher than that observed in those treated with chemotherapy alone [171].

Even though these results were encouraging, almost all trials were performed in the same center and mostly with a limited sample size. No other large studies were performed to confirm the results of Lissoni et al. Two studies by different research groups did not confirm prolonged survival after MEL administrations, the first in patients with brain metastases [172] and the second in non-small cell lung carcinoma [173]. MEL effect might be potentiated when applied as a part of multimodal treatment, like the Di Bella Method (MEL combined with somatostatin and biologically active compounds as retinoids, vitamins E, D3 and C and prolactin inhibitors) which showed positive results in lymphoma, leukemia, breast and prostate cancer patients [174,175,176,177,178]. However, these preliminary data have to be verified before this method can be recommended. Another important factor to establish is the pharmacokinetic properties of exogenous MEL in order to achieve an optimized clinical efficacy [179]. Nevertheless, with regards to its safety and positive reports in terms of alleviating the side-effects of chemotherapy or radiotherapy [180,181,182], systemic and/or topical application of MEL may at least improve the quality of life of cancer patients.

## 5. Pleiotropic Drugs and MEL in Cancer Prevention/Treatment

Preclinical and clinical reports support the hypothesis that MEL can improve the effect of other chemotherapeutics in several cancers. Our research group evaluated the effect of MEL combined with various agents including retinoids, non-steroidal anti-inflammatory drugs, statins and peroral antidiabetics in mammary cancer in vivo. In most cases, the effect of combinatory therapy was better in comparison with alone treatment. In the next sections, we focused on the latter three drugs that are used for metabolic disorders treatment, and we wrote a brief summary on the efficacy of these drug classes in cancer, together with available reports on their combination with MEL. The results of the relevant studies are summarized in Table S1.

### 5.1. NSAIDs

The history of the first representative of NSAIDs, which remains available to the present, dates back to 1899 when acetylsalicylic acid (aspirin) was introduced to the market by Bayer [183]. NSAIDs are widely prescribed for patients with coronary heart disease and rheumatoid arthritis. However, over the past three decades, epidemiological, clinical and experimental studies pointed to the significant anticancer effects of NSAIDs in various cancer types. In this regard, long-term administration of NSAIDs have been associated with reduced risk from cancer-related mortality and distant metastasis [184,185,186]. A recent meta-analysis demonstrated that NSAIDs are related to a significantly reduced risk of metastasis development (with the exception of lymph nodes), regardless of pre-diagnostic or post-diagnostic use [187]. Moreover, regular usage of NSAIDs is linked with a decreased risk of developing colonic adenomatous polyps and lower incidence of colorectal cancer [188,189] and several other neoplasia such as breast [190,191], ovary [192], lung [193], prostate [194], esophagus [195], gastric [196], endometrial [197] or pancreatic [198]. Based on the preclinical research, there is strong evidence about the chemopreventive efficacy of NSAIDs in cancer disease (review [199,200]). On the other hand, cancer chemoprevention using NSAIDs is not recommended, due to the potentially severe gastrointestinal, renal, and cardiovascular side effects that result from COX inhibition [201].

The anti-inflammatory effects of NSAIDs are attributed to the downregulation of cyclooxygenase (COX) enzymes that catalyze the conversion of arachidonic acid into prostaglandin H_2_, the precursor for the synthesis of eicosanoids, i.e., prostaglandins (PGs), prostacyclins and thromboxane A_2_. In addition, eicosanoids are critically important within the processes of homeostatic maintenance in organisms; it concerns with the gastrointestinal mucosa, blood clotting, regulation of blood flow and kidney functions [202]. There are two cyclooxygenase forms: constitutive COX-1 and the inducible COX-2 type; the latter is upregulated in inflammation and carcinogenesis. COX activation stimulates carcinogenesis through increased proliferation, invasiveness, angiogenesis, apoptosis inhibition and immune response modulation [203]. However, numerous studies provided evidence that anticancer effects can also be exerted through a COX-independent mechanism. COX-independent mechanisms of action include multiple pathways (such as PPARγ, PPARδ, RXRα, IKKβ, SERCA, CA IX/XII, Sp1, AMPK and gene expression of NAG-1 and 15-Lox-1) through direct molecular targets as well as epigenetic and post-transcriptional regulation responsible for the anticancer activities of NSAIDs (review [201]). COX-2 independent anticancer effects of NSAIDs comprise also decreasing of nuclear β-catenin levels and induction of β-catenin degradation, which could explain antiproliferative and pro-apoptotic activity of these drugs [204,205]. In addition, several studies with NSAIDs demonstrated that cyclic guanosine monophosphate phosphodiesterase (cGMP PDE) inhibition belongs among an important COX-independent mechanism suppressing β-catenin signaling pathway [206,207]. These data confirmed a mechanistic link between inhibition of cGMP PDE by NSAID and the blocking of Wnt/ β-catenin cell signaling.

#### NSAIDs and MEL

As mentioned above, the anticarcinogenic, chemopreventive and oncostatic potential of MEL has been reported in many in vitro and/or in vivo experimental studies against a variety of cancer types. A presumption exists that the combination of several anticancer substances with different mechanisms of efficacy can be more effective than application of individual substance. It is possible that the administration of NSAIDs in combination with MEL may elicit additive effects against cancer. Based on Pubmed and Scopus databases, there are only limited data describing anticancer efficacy of NSAIDs in combination with MEL. In our in vivo studies, we have evaluated the effect of indomethacin (non-selective COX inhibitor), nimesulide (preferential COX-2 inhibitor) and celecoxib (selective COX-2 inhibitor) as NSAIDs in combination with MEL in premenopausal chemically-induced mammary carcinogenesis in female rats (results are summarized in Table S1). In our first study, with 7,12-dimethylbenz(a)anthracene (DMBA)-induced carcinogenesis, MEL decreased tumor frequency and incidence versus controls; moreover, the combined chemoprevention with indomethacin manifested a slight additive effect when compared to indomethacin alone [208]. Interestingly, the combination of indomethacin and MEL reversed the oncostatic effect of indomethacin administered alone in N-methyl-N-nitrosourea (NMU)-induced rat mammary carcinogenesis [209]. We have evaluated tumor suppressive effects of nimesulide and MEL and their combination in both the NMU and DMBA models of rat mammary carcinogenesis. In the NMU study, nimesulide administered alone decreased tumor incidence and frequency and combined chemoprevention prolonged latency when compared to nimesulide alone. In the DMBA study, nimesulide alone was not effective, but a combination of nimesulide and MEL decreased the frequency compared to nimesulide alone [210]. Based on our results, it seems probable that the chemopreventive effect of indomethacine and nimesulide in rat mammary carcinogenesis depends on the type and dose of the carcinogenesis inducer, dose of the chemopreventive substance, the length and possibly also the way of administration and the time of the day when it is administered [210]. In our last study, celecoxib alone and in combination with MEL decreased tumor frequency. Combined treatment slightly improved the effect of single celecoxib as latency period increased. An interesting finding was the absence of tumors with comedonecrosis, which are more aggressive, in both goups with celecoxib [211].

A group in Spain conducted a preclinical study on the effects of celecoxib administered alone or in combination with MEL in Syrian hamsters with N-nitrosobis(2-oxopropyl)amine-induced pancreatic cancer [212]. The drugs were administered during induction, during postinduction and in both phases. MEL alone demonstrated a more potent anticancer activity compared to celecoxib regarding in that it reduced the oxidative stress and number of tumor nodules during the induction and the postinduction phases of pancreatic carcinogenesis and improved the survival of the animals. However, the combination of MEL and celecoxib administration showed a synergistic beneficial effect (restoring the survival of the animals) only during the postinduction phase (Table S1).

Taken together, the above studies suggested that the significance of MEL as an antineoplastic substance comes mainly from its combination with other oncostatic substances rather than from single administration.

### 5.2. Statins

The first statin, lovastatin was isolated from *Aspergillus terreus* by Merck in 1978, though it took several years of clinical investigation before it was approved for the market in the USA in 1987. At present, statins are the most-widely used drugs for the treatment of hypercholesterolemia. They had become a first choice in current prescribing practice and are pivotal in the primary and secondary prevention of cardiovascular disease [213,214]. Current preclinical studies have proven the pleiotropic properties of statins that can be useful for cancer therapy and prevention. Statins, influence mevalonate synthesis, inhibit dolichol-, farnesyl- and geranylgeranyl pyrophosphate production and block cancer from developing [215]. In vitro studies on various cell lines demonstrated the role of statins as growth inhibitors, by the induction of the G0/G1-arrest [216], G2/M arrest or cell death [217,218]. Proposed mechanisms for statin-mediated apoptosis include an upregulation of pro-apoptotic protein expression (Bax, Bim), together with decreased anti-apoptotic protein expression (Bcl-2), or activation of caspase-3, caspase-8 and caspase-9 [219,220]. In experiments of our group, the significant preventive effects of atorvastatin and simvastatin in rat mammary carcinogenesis were accompanied by an increase in the Bax/Bcl-2 ratio [221] and expression decrease of proliferating cell nuclear antigen (Ki67) [222] in mammary cancer in vivo. Angiogenesis plays an important role in tumor promotion and progression. Statins demonstrate significant anti-angiogenic and anti-metastatic activities by decreasing the vascular endothelial growth factor (VEGF) and matrix metalloproteinases expressions [223]. Fluvastatin in our preclinical study significantly downregulated VEGFR-2 expression in rat mammary carcinomas in vivo [224]. Moreover, statins were found to selectively slow proliferation of cancer stem cells through Rho-associated kinase 1 and focal adhesion kinase [225] or via inactivation of the Hippo/YAP/RhoA signaling in a mevalonate synthesis-dependent manner [226]. The promising anti-cancer effects of statins in preclinical research have stimulated investigations into their possible clinical implications as an anticancer agent in specific cancer types. There are several meta-analyses of clinical trials and observational studies available that have explored the potential benefits of statins in carcinogenesis. In some cases, promising results have been reported regarding their efficacy [227,228,229,230,231,232].

The oncostatic activities of statins summarized from preclinical and clinical research demonstrate their potential in the treatment of cancer patients. However, the doses of statins effective in the inhibition of proliferation and inducing the apoptosis are associated with higher toxicity in patients (myopathy, rhabdomyolysis and hepatotoxicity). For this reason, the use of statins as a monotherapy in cancer disease appears doubtful [233]. In order to reduce statinsʼ adverse effects, there are favored continuous low-dose drug clinical regimens. By using low statin doses during long-term administration, the inhibition of the mevalonate pathway might provide a more effective anti-cancer activity when combined with other chemotherapeutic agent [234]. Therefore, it seems likely that statins will be utilized within a combination with other anti-cancer drug in the treatment or prevention of cancer diseases. It could be foreseen that statins administered in combination with MEL will be more effective when compared with isolated drugs.

#### Statins and MEL

Regarding the evaluation of statins in combination with MEL, there are very limited data within oncological research. Recently, our group performed two experiments, where pravastatin or pitavastatin were combined with MEL in the chemopreventive/curative model of a chemically-induced rat mammary cancer model (results are summarized in Table S1). In our first study, only a slight non-significant anti-cancer effects of pravastatin alone was found, as tumor frequency decreased compared with the untreated control group. On the other hand, pravastatin combined with MEL markedly decreased tumor frequency (the most important parameter of rat mammary carcinogenesis) compared with control animals and also to pravastatin alone; tumor latency increased in the combination group compared to pravastatin alone [235]. The data from preclinical research have led us to hypothesize that statins and MEL might inhibit proliferation and angiogenesis and induce apoptosis in rat mammary tumor cells. Immunohistochemical analysis of tumor cells showed significant increase in the expression of caspase-3 and -7 after pravastatin and combined treatment in comparison with control group. Mammary cancer cell proliferation (KI67 expression) was higher in the pravastatin group but decreased in the combination of pravastatin and MEL when compared with pravastatin alone. Concerning the expression of VEGFR-2, there was a trend of decreased expression in tumor cells in both treated groups in comparison with the control group. Histopathological analysis confirmed an apparent shift in the rate of poorly differentiated (high-grade (HG)) and well-differentiated (low-grade (LG)) mammary carcinomas towards LG lesions in both groups; however, this effect was more pronounced after single pravastatin treatment. HG lesions are highly malignant or poorly differentiated, with loss of glanduliformity with solid growth, high grade of cellular atypia, high mitotic activity and abundant necrosis. On the other hand, LG tumors retain glandularity and show little variations in cellular changes and low mitotic activity and do not show comedonecrosis [235]. In our second experiment in the same model, pitavastatin did not show a significant anticancer effect, however, the combination of pitavastatin+MEL decreased tumor frequency and volume and slightly lengthened tumor latency compared to the control group. Moreover, compared to pitavastatin alone, a combination of pitavastatin+MEL decreased the tumor frequency. Immunohistochemical evaluation of carcinoma cells revealed a significant increase in the expression of caspase-3 and decrease in KI67 after pitavastatin and combined treatment. Additionally, the combination of pitavastatin and MEL decreased VEGF expression, which indicates suppression of angiogenesis [236]. The results from our laboratory showed that MEL has the potential to elevate the anti-cancer effects of statins. Although statins (mainly lipophilic) administered alone demonstrate significant anti-tumor effects, malignant cancer tissue as a highly dynamic structure manifests typical resistance to many forms of chemotherapy. Therefore, it is important to determine whether statins in combination with other chemotherapeutic agent (such as MEL) would provide a greater clinical benefit compared to single chemotherapy. Future, more prospective long-term follow-up studies should definitively answer the question about statinsʼ utilization within combination therapy in clinical oncology.

### 5.3. Peroral Antidiabetics

Diabetes incidence rises worldwide and type 2 diabetic patients constitute up to 90% of all cases [237]. For therapy, peroral antidiabetics are used predominantly; insulin is administered when peroral therapy is no longer effective. Patients with type-2 diabetes have a higher risk for almost all site-specific cancers, particularly liver and pancreatic cancer, with the exception of prostate cancer and melanoma [238,239]. Therefore, a great deal of attention is paid to antidiabetics with reported ability to modify carcinogenesis—metformin from biguanide group and glitazones (thiazolidinediones).

#### 5.3.1. Metformin

In 1950s, phenformin, buformin and metformin were synthesized and later approved for diabetes treatments; however, due to an increased risk of lactic acidosis, the first two were withdrawn from the market in the 1970s [240]. Metformin, though, has established its role and is now likely the most used therapy for type-2 diabetes all over the world. Biguanides were first suggested as a potential anticancer drugs as early as 1971 by Dilman [241] and preliminary in vivo research in late 1970s and early 1980s supported this expectation [242]; however, extensive research was not initiated until 2005, when results of a case-control study indicated that metformin use in patients with type 2 diabetes may reduce cancer risk [243]. Since then, a number of preclinical and clinical studies were conducted.

The key mechanism of antiproliferative action of metformin is contributed to mTOR inhibition via AMPK; however, metformin also inhibits cell proliferation through AMPK-independent mechanisms [244] and possesses the ability to target cancer stem cells and induce epigenetic changes which point to its pleiotropic beneficial effects [245]. In vitro results confirmed metformin’s oncostatic effects in a broad spectrum of neoplastic cells; we offer the readers some of these reports [246,247,248,249,250,251,252]. In vivo studies were performed almost exclusively in rodents, using different carcinogenic agent (chemocarcinogen, ionizing radiation, virus, spontaneous cancer) and various doses, routes and time-manner of metformin application. The main target organ was the mammary gland and colon (reviewed in [253]). Although inhibitory effects of metformin were not proved in all studies [254,255], none of the studies reported a stimulation of tumorigenesis. Variable outcomes, apart from differences in the dosing and manner of metformin administration, may be attributed to varying expression of genes related to pharmacokinetics and pharmacodynamics of metformin [255]. In addition, the effect may be modified by nutrition status; metformin toxicity against cancer cells increased in nutrient-poor conditions, which was proved both in vitro [256,257] and in vivo [256].

A recent comprehensive review summarizing the results of meta-analyses on the association between metformin use in diabetic patients and cancer risk reported decreased risk of gastric, liver, lung and endometrial cancer. Metformin significantly decreased the risk of advanced colorectal adenoma and improved colorectal cancer-related survival. Breast cancer incidence was not changed, however, overall survival was improved in metformin users [258]. Similarly, metformin did not significantly affect prostate cancer risk [259], however, it did improve recurrence-free [260] and overall survival [261]; overall survival was also improved in patients with pancreatic cancer [262]. Metformin as an adjuvant anticancer treatment in cancer patients with diabetes decreased all-cause mortality rates for colorectal cancer, endometrial cancer, breast cancer, prostate cancer and ovarian cancer and also cancer-specific mortality for breast cancer [263]. Available data justify the conclusion that metformin use is at least associated with reduced overall mortality of diabetic cancer patients.

Data on metformin use in non-diabetic cancer subjects are limited and include only a small number of patients. Except for one study which reported delayed response to chemotherapy in melanoma patients [264], metformin had beneficial effects. Brief metformin administration (in doses comparable to those used in diabetes treatment) to breast cancer patents before surgery decreased proliferation and increased apoptosis in tumor samples [265]; long-term administration as adjuvant therapy decreased risk of metastasis [266]. Short-term administration decreased colon carcinogenesis, as evidenced by suppressed aberrant crypt foci [267]. A decrease in proliferation after brief metformin treatment was also reported in endometrial [268] and prostate cancer patients [269]. This outcome supports the idea of a so called “metabolic rehabilitation” of cancer patients receiving metformin as adjuvant therapy suggested by professor Dilman [270]. Reported protective cardiovascular effects, reduction of inflammation, white adipose tissue remodeling, improvement of dyslipidemia and gut microbiota [271] contribute to the now generally accepted idea of an off-label use of metformin, not only in oncostatic therapy, but in a wide spectrum of pathologies.

##### Metformin and MEL

Research on simultaneous administration of metformin and MEL in cancer is surprisingly sparse (results are summarized in Table S1). In 2010, Anisimov et al. [272] reported inhibition of growth of transplantable mammary and Ehrlich carcinoma, though it did not influence spontaneous mammary tumor growth in mice. The same group reported inhibitory effects of metformin on benz(a)pyrene-induced skin carcinogenesis in mice [273,274] and an increase of the cytotoxic effect of paclitaxel on transplantable mammary tumors in mice after metformin and MEL combination [275]. The only available clinical report on the effect of this combination is a case report on adrenocortical carcinoma, a patient on maintenance therapy with metformin and MEL was reported to be free of disease some seven years post diagnosis [276] (Table S1).

Our group evaluated the effect of this combination in chemically-induced mammary cancer in rats and found more prominent inhibition of tumor growth and stimulation of apoptosis than in monotherapy in a DMBA model. However, it has to be emphasized that the effect of the combinatory therapy did not differ much from MEL monotherapy. Surprisingly, the proportion of HG tumors was not significantly changed [277]. No effect on tumor growth was recorded in the same model using NMU as tumor initiator single metformin. MEL alone reduced the proportion of HG tumors, but the combinatory therapy did not [278]. However, in these two experiments, we used a high-fat diet (10% of total fat), which likely interfered with the effects of chemopreventive agents (Table S1). The difference in impact on tumor progression might also arise from inhibition of cytochrome P450 family 1 enzymes involved in metabolic activation of DMBA in mammary epithelial and stromal cells [279] by MEL [280] whereas NMU act as a direct DNA-alkylating agent.

#### 5.3.2. Glitazones (Thiazolidinediones)

The first glitazone for diabetes treatment, troglitazone, was launched to market in 1997 (first in USA, then in Europe) but was withdrawn in Europe shortly after on the grounds of hepatotoxicity. The other derivates, pioglitazone and rosiglitazone, were introduced in USA in 1999 and the following year in Europe [281]. Later, however, concerns regarding its cardiovascular safety emerged and meta-analysis confirmed increased risk of myocardial infarction (though not overall cardiovascular mortality) in rosiglitazone users [282]. As a result, the European Medicines Agency recommended its suspension from the market. It is currently still available in the USA but with several restrictions. Pioglitazone has remained the only available glitazone in Europe, however, it was withdrawn from the market in France and Germany in 2011 due to a suspected risk of bladder cancer. This concern still persists, as the conclusions of recent meta-analyses are contradictory [283,284,285]. A third commercially available glitazone analogue, lobeglitazone, was approved for use in Korea in 2013. A number of compounds have been developed, including dual PPAR (peroxisome-proliferator-activated receptors) agonists (PPARα/γ, PPARα/δ and PPARδ/γ) and pan-PPAR agonists or selective modulators. Unfortunately, many of them have been discontinued during the clinical research stage due to safety and tolerability issues such as weight gain, edema, congestive heart failure and bone fracture [286].

Glitazones exert their effects through the modulation of PPARγ, one of three subtypes of transcription factors from nuclear hormone receptor superfamily. The other two involve PPARα and PPARδ (also known as PPARβ [287]). PPARγ may be activated by natural ligands including fatty acids and synthetic ligands like glitazones. After the formation of a heterodimer with retinoid X receptor (RXR), this complex then binds to PPAR-responsive elements in DNA [288]. PPAR target genes are involved in the regulation of adipocyte differentiation and adipogenesis, energy homeostais, glucose and lipid metabolism and also in inflammation [289]. Expression of PPARγ was detected in various cancers [290]. Glitazones may inhibit cell growth and proliferation through the decrease in circulating insulin and modulation of the key pathways of the insulin/IGF axis (e.g., PI3K/mTOR, MAPK, and GSK3-β/Wnt/β-catenin cascades), which regulate cancer cell survival, cell reprogramming and differentiation [291]. PPARγ activation is regarded as the key mechanism of anticancer effects of glitazones. PPARγ agonists, in most cases, inhibited proliferation and growth of experimental breast, lung, gastrointestinal, liver, pancreatic, ovarian, testicular and urinary system cancers [292,293,294,295,296]. Antitumor properties independent of PPARγ activation were reported too, including the regulation of differentiation, inflammation and apoptosis; however, it is not always possible to determine whether an effect that is independent of PPARγ-regulated transcriptional control is also independent of the presence of PPARγ protein [297,298].

Adjuvant treatment with glitazones increased the effect of chemotherapy and radiotherapy [299]. However, neither expression [300] nor activation of PPARγ [301] was invariably correlated with a positive outcome in cancer.

Unlike preclinical data, which showed oncostatic effect in wide range of neoplasms, results of meta-analyses on the impact of glitazone therapy (rosiglitazone or pioglitazone) in diabetic patients showed the inverse relation only with liver and colorectal cancer [302,303]. The risk of breast cancer reported previously [304] was not confirmed in the recent meta-analysis by Du et al. [305], however, diabetic women with HER2-positive breast cancer showed lower breast cancer specific mortality when treated with glitazones [306]. No association was found with lung, prostate and pancreatic cancer [262,302]. The slight increases of bladder cancer risk regarding pioglitazone use, particularly in higher doses and long-term administration is still being discussed. However, firstly, bladder cancer is more likely to occur in patients with diabetes [307] and secondly, pioglitazone is recommended as a third-line therapy for type 2 diabetes. Thus, it is being prescribed to patients with more advanced forms of the disease with a higher possibility of metabolic and other complications which could be another confounding factor for the increased bladder cancer risk [284]. Another adverse effect is the increased risk of bone fractures in women, both after pioglitazone and rosiglitazone [308]. Therefore, doubts and safety concerns about the role of glitazones in diabetes type 2 management persist. On the other hand, it is important to point to other beneficial effects of glitazones as favorable fat distribution, amelioration of renal and liver functions and also, in the case of pioglitazone, improvement of cardiovascular profile in patients with diabetes [309]. Moreover, severity and duration of diabetes, related comorbidities and other risk factors such as exposure to environmental carcinogens, must be considered in evaluation of any adverse effects of antidiabetic therapy.

Due to safety issues, reports on glitazone therapy in nondiabetic cancer patients are scarce and based on a limited numbers of patients, mostly in advanced stages of the disease, and when glitazones were administered for short time periods. In liposarcoma patients, troglitazone decreased proliferation in tumor biopsies [310], however, no effect was found after rosiglitazone treatment [311]. Troglitazone treatment did not led to tumor response and improved survival in patients with metastatic colon cancer [312]. Rosiglitazone had no redifferentiating effect in patients with thyroid cancer [313] and did not prolong the time to disease progression in patients with prostate cancer [314]. No effect was found in women with breast cancer after rosiglitazone [315] or troglitazone treatment [316]. Combinatory therapy may improve the outcome of standard chemotherapy: pioglitazone and rofecoxib combination showed some response in patients with metastatic melanoma and soft tissue carcinoma [317], malignant vascular tumors [318] and in glioma patients [319]. On the other hand, rosiglitazone in combination with metformin did not improve the efficacy of exemestane therapy in non-diabetic postmenopausal women [320] and in combination with bexarotene did not result in an objective response in patients with refractory solid tumors [321]. Preliminary data indicate that pioglitazone may improve the effect of imatinib in chronic myeloid leukemia [322]. Appropriate conclusion regarding the role of glitazones in cancer treatment cannot be made due to the limitations of available studies.

##### Glitazones and MEL

MEL enhanced troglitazone-induced apoptosis in the MDA-MB-231 breast cancer cell line [323]. Our working group has investigated the effect of pioglitazone and combination pioglitazone+MEL in a mammary cancer model in rats. In this experiment, we used, unlike in our previous research where pioglitazone inhibited the growth of NMU-induced tumors [324], a diet with higher fat content in order to better reflect the situation in human population. Although parameters of tumor growth were not significantly changed, pioglitazone alone decreased proportion of HG tumors and increased apoptosis in mammary cancer cells. MEL potentiated the effect of pioglitazone as evidenced by decrease in tumor frequency and further reduction in HG/LG ratio (Table S1, [325]).

### 5.4. Retinoids and MEL

The oncostatic abilities of retinoids are well-known, although some adverse effects have been reported too [326]. In this review, we did not bring the summary on the mechanism of action and effects of retinoids and rexinoids in preclinical and clinical cancer research; we refer readers to several excellent reviews, e.g., [327,328,329,330]. Nonetheless, we wished to highlight the ability of MEL to potentiate their oncostatic effects; thus, we included the results of relevant animal studies (Table S1) which clearly point to this capability [331,332,333,334,335,336].

## 6. Conclusions

Now, sixty years after MEL discovery, extensive research has brought clear evidence on its beneficial effects in modulation of cancer progression and even if data are not always cohesive, none point to the stimulation of malignant growth. An increase in autoimmune proinflammatory states and/or metabolic disturbances and a decline in physiological MEL levels with age on one side and increase in cancer rates in elderly subjects on the other supports the hypothesis that the addition of exogenous MEL to therapy of above-mentioned states by non-steroidal anti-inflammatory drugs/statins/antidiabetics may modulate a cancer risk as well. As malignant transformations, from initiation to clinical manifestation, may take years or even decades, long-term application of MEL should be performed. Available experimental data on the efficacy of the combination of MEL with pleiotropic drugs are limited and human data are practically non-existent. However, preliminary results and safety of MEL provide a rationalization of further, more systematic research.

## Supplementary Materials

The following are available online at http://www.mdpi.com/1422-0067/19/12/3910/s1, Table S1: Oncostatic effects of MEL added to treatment with chosen pleiotropic drugs/chemotherapeutics in vivo and in human trials.
