# Supplementary material for: Melatonin May Increase Anticancer Potential of Pleiotropic Drugs
Source: Int J Mol Sci. 2018 Dec 6;19(12):3910. doi: 10.3390/ijms19123910 (PMC6320927; doi:10.3390/ijms19123910)
Supplement: Supplementary file 1 [file ijms-19-03910-s001.pdf]

Table S1. Oncostatic effects of melatonin (MEL) added to treatment with chosen pleiotropic drugs/chemotherapeutics in vivo and in human trials.

Species, strain/study	Tumor site	Initiating agent	Chemopreventive/ chemotherapeutic agent	Effect on tumor growth: chemopreventive agent(s)	Effect on tumor growth: MEL	Effect of combination with MEL compared to single agent	Reference
Female Sprague-Dawley rats	Mammary gland	DMBA	Indomethacin	↓ tumor incidence, frequency and volume<br>↑ latency	↓ tumor incidence and frequency	↓ tumor incidence (ns)	[208]
		NMU		**↑ tumor volume**<br>**↑ tumor incidence (ns)**<br>**↓ latency (ns)**	↓ tumor volume<br>↓ tumor frequency (ns)	↑ latency (ns)<br>↓ tumor volume (ns)	[209]
		DMBA	Nimesulide	≈	↓ tumor incidence<br>↓ tumor frequency and volume (ns)	↓ tumor volume<br>↓ tumor frequency (ns)	[210]
		NMU		↓ tumor incidence and frequency<br>↓ tumor volume	**↑ tumor volume**<br>↓ tumor incidence (ns)	↑ latency (ns)	
			Celekoxib	↓ tumor frequency<br>↓ tumor incidence (ns)	↓ tumor frequency (ns)<br>↑ latency (ns)	↑ latency (ns)	[211]
Male Syrian hamsters	Pancreas	BOP		↓ tumor nodules<br>↑ survival<br>↓ oxidative stress	↓ tumor nodules<br>↑ survival<br>↓ lung metastases<br>↓ oxidative stress	↓ oxidative stress	[212]
Female Sprague-Dawley rats	Mammary gland	NMU	Pravastatin	↑ apoptosis<br>↓ tumor volume<br>↓ HG tumor proportion<br>**↑ proliferation**<br>↓ tumor frequency (ns)	-	↑ apoptosis<br>↓ proliferation<br>↓ tumor frequency and volume<br>↑ latency<br>↓ tumor incidence (ns)	[235]
			Pitavastatin	↑ apoptosis<br>↓ proliferation<br>↓ tumor volume<br>↑ latency (ns)	-	↓ VEGF expression<br>↓ tumor frequency (ns)	[236]
Male SHR mice	Skin	BP	Metformin	↓ tumor incidence, frequency and volume<br>↑ latency and survival	↓ tumor incidence, frequency and volume<br>↑ latency and survival	↓ tumor incidence,	[273]
Female SHR mice				↓ tumor frequency and volume	↓ tumor volume<br>↓ tumor incidence (ns)<br>↑ latency (ns)	↓ tumor incidence (ns)	[274]

Species, strain/study	Tumor site	Initiating agent	Chemopreventive/ chemotherapeutic agent	Effect on tumor growth: chemopreventive agent(s)	Effect on tumor growth: MEL	Effect of combination with MEL compared to single agent	Reference
Clinical study		Melanoma treated with dacarbazine		**Delayed response to chemotherapy**		-	[264]
Case-report	Adrenals	Adrenocortical carcinoma		**-**		7-year remission post diagnosis	[276]
Female FVB/N HER2/neu mice	Mammary gland	Spontaneous		↑ latency<br>↑ survival	↑ survival	≈	[272]
Male FVB/N mice	Mammary carcinoma	HER2/neu transplants		≈	≈	↓ tumor volume<br>↑ survival	
Male SHR mice		Ehrlich carcinoma transplants		-	-	↓ tumor volume<br>↑ survival (ns)	
Female FVB/N mice		HER2/neu transplants		↓ tumor incidence<br>↑ survival (ns)	≈	-	[275]
			Paclitaxel	↓ tumor incidence<br>↑ survival (ns)		↓ tumor incidence<br>↑ survival (ns)	
			Metformin<br>+ paclitaxel	↓ tumor incidence<br>↑ survival (ns)		↓ tumor incidence (ns)<br>↑ survival (ns)	
Female Sprague-Dawley rats	Mammary gland	NMU+high-fat diet	Metformin	↓ HG tumor proportion<br>↓ tumor volume	↓ HG tumor proportion<br>↓ proliferation<br>↓ incidence (ns)	≈	[278]
		DMBA+high-fat diet		↓ tumor volume<br>↑ apoptosis (ns)<br>↓ tumor incidence (ns)	↑ apoptosis<br>↓ tumor volume<br>↓ tumor incidence and frequency (ns)<br>↑ latency (ns)<br>↓ HG tumor proportion (ns)	↑ apoptosis<br>↓ tumor incidence and frequency (ns)<br>↓ HG tumor proportion (ns)	[277]
		NMU+high-fat diet	Pioglitazone	↑ apoptosis<br>↓ HG tumor proportion	↑ apoptosis<br>↑ latency (ns)	↓ tumor incidence and frequency (ns)<br>↓ HG tumor proportion (ns)	[325]
		DMBA	Retinyl acetate	↓ tumor incidence and frequency<br>↑ latency	↓ tumor frequency<br>↑ latency (ns)	↓ tumor frequency (ns)	[331]
		NMU		↑ latency (ns)	↑ latency<br>↓ tumor incidence (ns)	↓ tumor incidence and frequency	[332]

Species, strain/study	Tumor site	Initiating agent	Chemopreventive/ chemotherapeutic agent	Effect on tumor growth: chemopreventive agent(s)	Effect on tumor growth: MEL	Effect of combination with MEL compared to single agent	Reference
			9-cis-retinoic acid	↓ tumor incidence, frequency and volume (ns)	-	↓ tumor incidence (ns)	[333]
				↓ tumor incidence, frequency and tumor area	↓ tumor incidence, frequency and tumor area	↓ tumor incidence, frequency and tumor area	[334]
				↓ ERα mRNA expression<br>↓ tumor frequency (ns)	↓ ERα mRNA expression<br>↓ tumor frequency and volume (ns)	↓ tumor frequency and volume<br>↑ apoptosis<br>↓ ERα mRNA expression	[335]
			Bexarotene	↓ tumor frequency<br>↓ HG tumor proportion<br>↓ tumor incidence (ns)	-	↓ tumor incidence<br>↓ frequency (ns)	[336]

Abbreviations: BOP, N-nitrosobis(2-oxopropyl)amine; BP, benz(a)pyrene; DMBA, 7,12-dimethylbenz(a)anthracene; ER, estrogen receptor; HG, high-grade; NMU, N-methyl, N-nitrosourea; ns, non-significant; -, no results; ≈, no effect; ↓, decrease in parameter; ↑, increase in parameter. Boldface indicate undesirable effect.
